# Detecting Brain Activity Following a Verbal Command in Patients With Disorders of Consciousness

**DOI:** 10.3389/fnins.2019.00976

**Published:** 2019-09-13

**Authors:** Fuyan Wang, Nantu Hu, Xiaohua Hu, Shan Jing, Lizette Heine, Aurore Thibaut, Wangshan Huang, Yifan Yan, Jing Wang, Caroline Schnakers, Steven Laureys, Haibo Di

**Affiliations:** ^1^International Unresponsive Wakefulness Syndrome and Consciousness Science Institute, Hangzhou Normal University, Hangzhou, China; ^2^Department of Radiology, The Affiliated Hospital of Hangzhou Normal University, Hangzhou, China; ^3^Department of Rehabilitation, Hangzhou Wujing Hospital, Hangzhou, China; ^4^INSERM, U1028, CNRS, UMR5292, Auditory Cognition and Psychoacoustics Team, Lyon Neuroscience Research Center, Lyon, France; ^5^Coma Science Group, GIGA-Research, CHU University Hospital of Liège, Liège, Belgium; ^6^Department of Neurosurgery, University of California, Los Angeles, Los Angeles, CA, United States

**Keywords:** vegetative state, unresponsive wakefulness syndrome, minimally conscious state, hand raising, disorders of consciousness, functional magnetic resonance imaging

## Abstract

**Background:**

The accurate assessment of patients with disorders of consciousness (DOC) is a challenge to most experienced clinicians. As a potential clinical tool, functional magnetic resonance imaging (fMRI) could detect residual awareness without the need for the patients’ actual motor responses.

**Methods:**

We adopted a simple active fMRI motor paradigm (hand raising) to detect residual awareness in these patients. Twenty-nine patients were recruited. They met the diagnosis of minimally conscious state (MCS) (male = 6, female = 2; *n* = 8), vegetative state/unresponsive wakefulness syndrome (VS/UWS) (male = 17, female = 4; *n* = 21).

**Results:**

We analyzed the command-following responses for robust evidence of statistically reliable markers of motor execution, similar to those found in 15 healthy controls. Of the 29 patients, four (two MCS, two VS/UWS) could adjust their brain activity to the “hand-raising” command, and they showed activation in motor-related regions (which could not be discovered in the own-name task).

**Conclusion:**

Longitudinal behavioral assessments showed that, of these four patients, two in a VS/UWS recovered to MCS and one from MCS recovered to MCS+ (i.e., showed command following). In patients with no response to hand raising task, six VS/UWS and three MCS ones showed recovery in follow-up procedure. The simple active fMRI “hand-raising” task can elicit brain activation in patients with DOC, similar to those observed in healthy volunteers. Activity of the motor-related network may be taken as an indicator of high-level cognition that cannot be discerned through conventional behavioral assessment.

## Introduction

During clinical assessment of patients with disorders of consciousness (DOC), overt behaviors may be ambiguous or absent (Schnakers et al., 2009). In these cases, functional neuroimaging paradigms can assist conventional behavioral assessment (Davey et al., 2000; Coleman et al., 2009; Stender et al., 2014). However, without a full understanding of the neural correlates of consciousness, even a near-to-normal activation elicited by passive sensory stimulation cannot prove that these patients are aware. All that can be deduced is that a certain brain region is still able to process the correlative sensory stimuli. Recent functional magnetic resonance imaging (fMRI) studies have demonstrated preserved conscious awareness in some patients meeting the clinical criteria for a unresponsive wakefulness syndrome (UWS) (Laureys et al., 2010), using tasks that express “volitional” aspects of behavior, such as communicating “yes”/“no” responses by using mental imagery paradigms of “playing tennis” and navigating in one’s house (Giacino et al., 2002; Boly et al., 2007; Monti et al., 2010). Although a predictable activation in response to the instruction to perform a mental imagery task would infer consciousness in non-communicative patients, on account of the task execution difficulty (Comte et al., 2015), patients with a positive response are rare (Latronico, 2016).

This is not surprising as these motor imagery paradigms require patients to process two different mental tasks of high cognitive load, which can be difficult and tiresome to perform for patients in so severe state (Beukema et al., 2016). A more extensive review on the subject of active fMRI paradigms can be found in related review articles (Owen, 2013, 2014). For some unresponsive patients without overt behavior like hand raising, for example, the volition of raising their hand may still exist and go unnoticed at the bedside. In that respect, clinicians are faced with false-negative diagnostic incidence. We aimed to assess the usefulness of a simpler task to increase the sensitivity of the neuroimaging methodology and improve diagnostic precision.

Following motor-related commands, such as “raise your hand”, are commonly observed markers of consciousness leading to the diagnosis of a minimally conscious state (MCS) (Giacino et al., 2002). We hypothesize that this command seems more straightforward for patients to understand and perform with higher reliability (Bekinschtein et al., 2011). Here, we used fMRI to detect the brain activation in patients with DOC when they were aurally instructed to physically raise their hand. Studies in healthy subjects have shown that hand movement execution involves activations in the supplementary motor cortex (SMA), the primary motor cortex (PMC/M1), and the cerebellum (Pfurtscheller and Neuper, 1997; Fins and Schiff, 2000; Nachev et al., 2008; Strick et al., 2009). Based on comparable brain activation patterns and other evidence clusters induced by this experimental manipulation, we hypothesized that mind execution of hand raising in patients with DOC could be differentially inferred.

## Materials and Methods

### Participants

This experiment was performed in 31 patients with DOC. All patients enrolled were verified suitable for MRI scanning by experienced neurologists. Patients with severe head injury history, reported neurological disorders, brain injury less than a month before scanning were excluded. We also used the auditory startle reflex test to exclude patients who could not shut their eyes while listening to a sudden big noise produced by clapping (above patient’s head, out the sight of the patient, similar to CRS-R auditory startle assessment). Due to uncontrolled head movements, two patients were excluded during data preprocessing. Of the 29 remaining patients, 16 were suffering from traumatic brain injury, 7 had DOC caused by anoxic brain injury, and 6 by cardiovascular accident. The patients’ ages ranged from 8 to 63 years (mean age = 39 ± 14 years), and the time of the study ranged from 1 to 154 months post-injury (mean time = 15 months). To make a reliable behavioral diagnosis, each patient was evaluated using the Coma Recovery Scale–Revised (CRS–R) (Giacino et al., 2004; Di et al., 2017; Zhang et al., 2019). Based on extensive and repeated clinical testing (at least three times, the last CRS-R assessment was performed several hours before MR scanning), 21 patients were classified as vegetative state/unresponsive wakefulness syndrome (VS/UWS) (male = 17, female = 4), 8 with MCS (male = 6, female = 2). The clinical data of these patients are summarized in Table 1. Fifteen healthy subjects were included as a control group (9 females, age range 18–27 years, mean age 24 years); none reported any history of head injury, neurological or psychiatric disorders.

**TABLE 1:** Shows the characteristic data and the follow-up diagnosis at 3, 6, and 12 months of the patients with disorders of consciousness.

**No.**	**Sex/age, y**	**Diagnosis**	**Cause**	**MPI**	**3 Months diagnosis**	**6 Months diagnosis**	**12 Months diagnosis**
VS1	M/20	VS	TBI	20	VS	VS	MCS
VS2	M/47	VS	Anoxic brain injury	2	VS	VS	VS
VS3	M/20	VS	TBI	24	MCS	MCS	MCS
VS4	M/31	VS	TBI	6	MCS	MCS	MCS
VS5	M/48	VS	TBI	2	MCS	Died	Died
VS6	M/54	VS	CVA	2	VS	VS	Died
VS7	M/27	VS	TBI	1	EMCS	EMCS	EMCS
VS8	M/28	VS	TBI	60	MCS	MCS	MCS
VS9	F/31	VS	TBI	2	MCS	MCS	MCS
VS10	F/8	VS	TBI	3	MCS	MCS	MCS
VS11	M/36	VS	Anoxic brain injury	2	VS	VS	VS
VS12	M/33	VS	Anoxic brain injury	154	VS	VS	VS
VS13	M/63	VS	TBI	18	VS	MCS	MCS
VS14	F/45	VS	TBI	3	MCS	MCS	MCS
VS15	M/45	VS	TBI	12	VS	VS	VS
VS16	M/23	VS	Anoxic brain injury	17	VS	VS	VS
VS17	M/20	VS	Anoxic brain injury	7	VS	VS	VS
VS18	M/43	VS	CVA	3	VS	VS	VS
VS19	M/21	VS	TBI	6	VS	VS	VS
VS20	F/38	VS	Anoxic brain injury	3	VS	VS	VS
VS21	M/54	VS	Anoxic brain injury	2	VS	VS	VS
MCS1	M/32	MCS	TBI	2	EMCS	EMCS	EMCS
MCS2	F/55	MCS	CVA	24	MCS-	MCS-	MCS +
MCS3	M/50	MCS	CVA	14	MCS	MCS	MCS
MCS4	F/59	MCS	CVA	5	MCS	MCS	MCS
MCS5	M/62	MCS	CVA	17	MCS	MCS	MCS
MCS6	M/37	MCS	TBI	4	EMCS	EMCS	EMCS
MCS7	M/42	MCS	TBI	24	EMCS	EMCS	EMCS
MCS8	M/50	MCS	TBI	9	MCS	MCS	MCS

*VS, vegetative state; MCS, minimally conscious state; EMCS, emergence from minimally conscious state; TBI, traumatic brain injury; CVA, cerebrovascular accident; MPI, months post-ictus. M, male; F, female.*

To examine the prognostic value of this fMRI paradigm and stability of diagnoses, longitudinal behavioral assessments were conducted using the CRS–R at the time of scanning and at 3, 6, and 12 months after fMRI acquisitions; the follow-up data are summarized in Table 1.

Written informed consent was obtained from the legal representatives of all the patients and all the healthy volunteers. The study was approved by the Ethics Committee of Hangzhou Normal University School of Medicine, Hangzhou, China.

### Imaging Data Acquisition and Analysis

Using GoldWave software (GoldWave Inc.), we digitally recorded the voice of a first-degree family member calling the patients’ own name (SON-FV; subject own) and the command “raise your hand”. fMRI scanning was performed using a block design, with six active blocks and seven baseline blocks for each run. Each active block lasted 12 s and included the command “raise your hand” seven times (each one lasted 1 s), and each baseline block lasted 18 s, during which only the attenuated machine noise was presented. The auditory stimuli were presented through MRI-compatible noise-attenuated headphones (Resonance Technology, Inc., Los Angeles, CA, United States).

Data were collected using a 1.5T General Electric Sigma Horizon MRI system (GE Medical Systems, Milwaukee, WI, United States). First, 22 axial anatomic images were collected using a T1-weighted spin echo sequence [repetition time (TR) = 500 ms, echo time (TE) = 9 ms, field of view (FOV) = 240 × 240 mm, slice thickness = 5 mm, skip = 1 mm, matrix = 256 × 256, with the resolution of three dimensions of one voxel: *x* = 0.9375 mm, *y* = 0.9375 mm, *z* = 6 mm]. Next, 120 images per slice were acquired using a gradient echo planar imaging (TR = 3000 ms, TE = 60 ms, matrix = 64 × 64, with the resolution of three dimensions of one voxel: *x* = 3.75 mm, *y* = 3.75 mm, *z* = 6 mm). Finally, a fast spoiled gradient recalled sequence (TR = 27 ms, TE = 6 ms, FOV = 240 × 240 mm, matrix = 256 × 256, with the resolution of three dimensions of one voxel: *x* = 1.3 mm, *y* = 0.9375 mm, *z* = 0.9375 mm) was used in a sagittal plane to collect three-dimensional images covering the entire brain volume. The imaging procedures and parameters were similar to those of our previously published studies (Di et al., 2007; Wang et al., 2015).

Analysis of Functional NeuroImages (AFNI) software (Schiff et al., 2000) was used for data preprocessing and analysis. After correcting for two- and three-dimensional head motion, the functional images were smoothed using an isotropic gaussian kernel (full width at half maximum = 6 mm). We then performed multiple linear regression analysis (using the 3dDeconvolve program in AFNI) to further correct for head movement artifacts (six estimated motion-induced time series used as regressors of no interest), to generate activation maps and identify SON-FV and “raise your hand”-induced Blood Oxygenation Level Dependent (BOLD) signal increases. Significance values were calculated to test the fit between the estimated response and the observed signal for each voxel, and they were corrected for multiple comparisons by combination of individual voxel probability and minimum cluster size at *t* > 2 (*p* < 0.05, corrected). In addition, to avoid further false-negative results, a cluster size of 10 voxels was used as the other additional threshold, which was similar to another study (Rodriguez Moreno et al., 2010). Accurately identifying these cortical areas (the auditory cortex and motor-related cortex) in deformed brains may be difficult; therefore, special care was taken to segment the auditory cortices and motor-related cortices of each patient by repeatedly and simultaneously checking the anatomic landmarks in three orthogonal cross-sectional views (axial, coronal, and sagittal) of the individual high-resolution three-dimensional brain images. The significantly activated voxels were then superimposed on the anatomically defined patients’ brains. In order to check the location of the activated area in the positive command-following patients, we checked both the original images and the images transformed to standard space cortices (the Talairach space available in the AFNI software). The data processing procedures have also been used in previous published studies (Wang et al., 2015).

## Results

**FIGURE 1:**
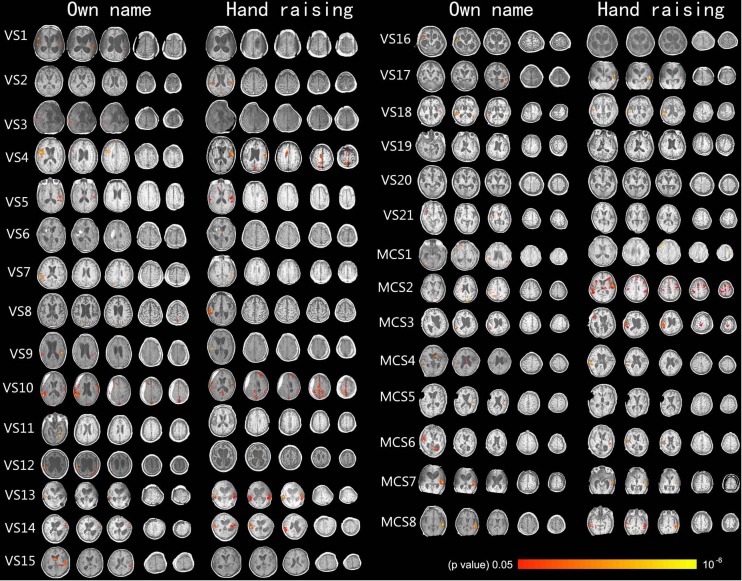
Shows the activation of the auditory cortex caused by own name stimulation and activation of the motor-related cortex caused by the hand-raising command in 29 DOC patients (axis view, *p* < 0.05, corrected).

**FIGURE 2:**
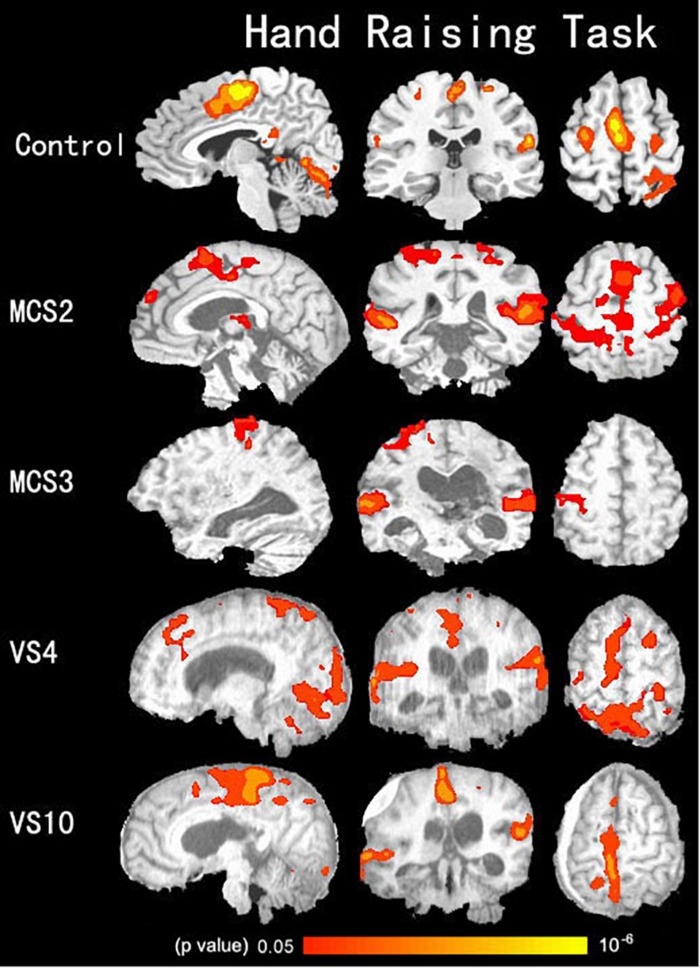
Shows activation in the auditory cortex, the motor-related cortex (the supplementary motor cortex, primary motor cortex, anterior cingulated cortex, and the cerebellum) during the hand-raising task in two VS/UWS patients, two MCS patients, and 15 healthy volunteers in the control group (*p* < 0.05, corrected).

**FIGURE 3:**
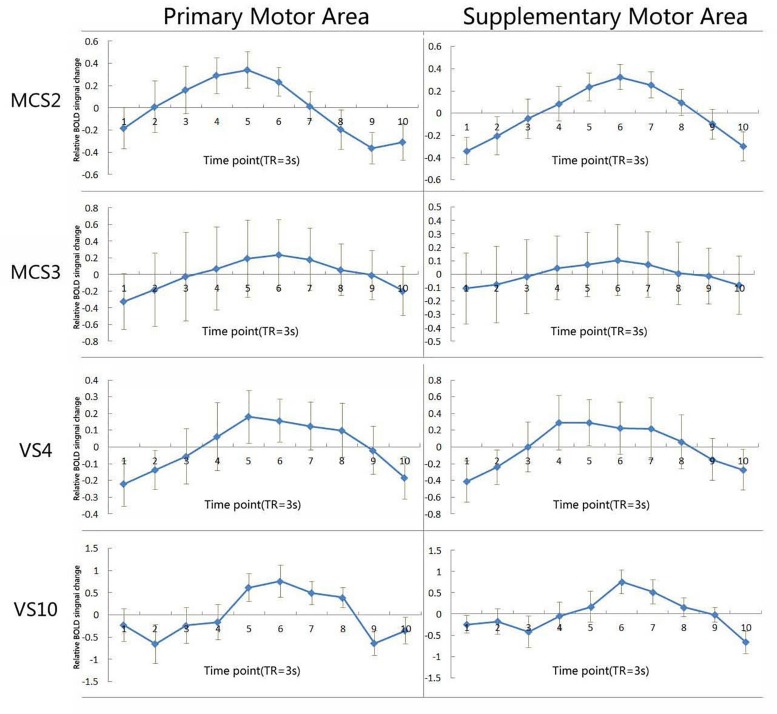
Shows the average time course of the voxels activated in the primary motor area and the supplementary motor area when exposed to the hand-raising command in two VS/UWS patients, and two MCS patients. The I bars represent standard errors. BOLD denotes blood-oxygenation-level–dependent.

Of the 29 enrolled DOC patients, four showed activation in motor-related areas while performing the hand-raising task. All activation maps are summarized in Figure 1. For these four patients, two were in VS/UWS (VS4 and VS10), two were in MCS (MCS2 and MCS3), as shown in Figure 2. VS10 was an 8-year-old girl, who strictly fulfilled the criteria for VS/UWS with clinical CRS-R assessment. She showed significant activation in the SMA, the PMC(M1), the anterior cingulated cortex (ACC) and the cerebellum during the “hand-raising” period. The brain activation areas related to hand raising task of all DOC patients are listed in Table 2. Her neural responses could not be distinguished from those observed in 15 healthy volunteers performing the same imagery tasks. VS10 had been scanned twice, and her motor network showed activation in the second scan but no activation during the first. The two patients in MCS showed similar activation comparable to controls in motor areas during the hand-raising task. In none of the four patients could we observe an actual hand-raising response in the scanner. As to presentation of their own name, no activation was found in the SMA or M1, although some showed activation in the cerebellum, which is similar to the situation in healthy volunteers (Schutter and van Honk, 2005; Stoodley, 2012). The SMA and M1 time series of these four patients during the hand-raising paradigm are shown in Figure 3. Longitudinal behavioral assessments showed that, of these four patients with positive activation, two in a VS/UWS evolved to MCS and one from MCS evolved to MCS+. In patients with no motor-related activation in hand raising task, 6 in a VS/UWS and 3 in a MCS showed recovery in follow-up procedure.

**TABLE 2:** Shows the brain activation area related to the hand-raising task in all patients with disorders of consciousness.

**Hand raising task**					

**Paitent**	**PMC**	**SMA**	**ACC**	**Cerebellum**	**Network status**
VS1	×	×	×	×	Absent
VS2	×	×	×	×	Absent
VS3	×	×	×	×	Absent
VS4	√	√	√	√	Complete
VS5	×	×	×	×	Absent
VS6	×	×	×	×	Absent
VS7	×	×	×	×	Absent
VS8	×	×	×	×	Absent
VS9	×	×	×	×	Absent
VS10	√	√	√	√	Complete
VS11	×	×	×	×	Absent
VS12	×	×	×	×	Absent
VS13	×	×	×	×	Absent
VS14	×	×	×	×	Absent
VS15	×	×	×	×	Absent
VS16	×	×	×	×	Absent
VS17	×	×	×	×	Absent
VS18	×	×	×	×	Absent
VS19	×	×	×	×	Absent
VS20	×	×	×	×	Absent
VS21	×	×	×	×	Absent
MCS1	×	×	×	×	Absent
MCS2	√	√	√	×	Partial
MCS3	√	×	×	×	Partial
MCS4	×	×	×	×	Absent
MCS5	×	×	×	×	Absent
MCS6	×	×	×	×	Absent
MCS7	×	×	×	×	Absent
MCS8	×	×	×	×	Absent

*VS, vegetative state; MCS, minimally conscious state; PMC, primary motor cortex; SMA, supplementary motor area; ACC, anterior cingulate cortex; Sign ‘√’ represents observed activation in certain brain area; Sign ‘×’ represents certain brain area has no reaction to verbal command.*

## Discussion

In this study, hand raising was used as an active fMRI paradigm to determine the incidence of undetected awareness in 29 patients with DOC. We identified two patients in VS/UWS, two in MCS, who could modulate their brain activity reliably and repeatedly to the hand-raising instruction. Interestingly, for these four patients, behavioral changes using the CRS-R scores were observed at the 3 and 6-month follow-up: the two patients in VS/UWS evolved to MCS, and one patient initially in MCS showed a CRS-R score similar to a conscious patient. Basing on demographic and clinical behavioral assessment information, no difference could be found between VS and MCS patients, neither before nor after the follow-up procedure (improved and not improved outcome, all Chi-squared values > 1). This follow-up result suggested that the activation elicited by hand raising might have a prognostic value in patients with DOC, however, the inherent difficulty of active fMRI paradigm limited its implemented potential when predicted recovery outcome of DOC patients.

**FIGURE 4:**
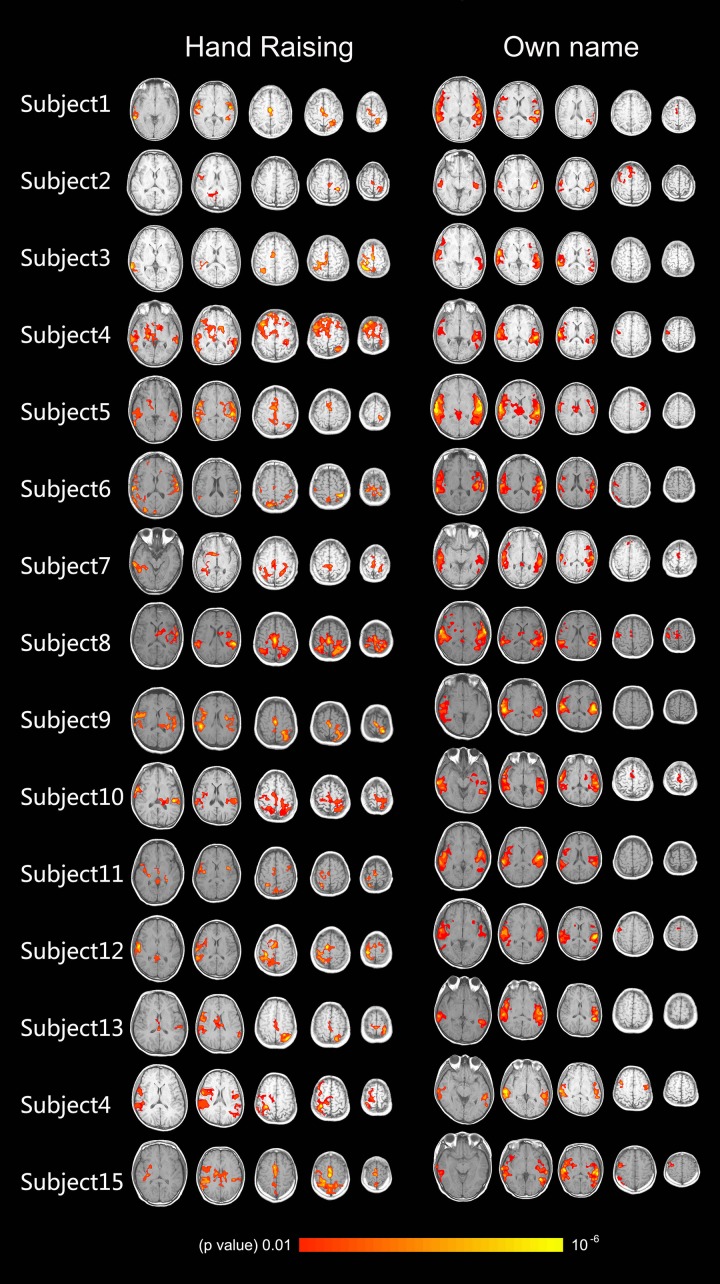
Shows the activation of the auditory cortex caused by own name stimulation and activation of the motor-related cortex caused by the hand-raising command in 15 healthy volunteers (axis view, *p* < 0.01, corrected).

The successful execution of the hand-raising task implies that the healthy subjects and patients first had the ability to comprehend the instructions, and then were able to initiate the hand movement, brain activation details of control group are listed in Table 3 and Figure 4. Such responses were hence considered volitional and indicative of preserved conscious awareness. The SMA, M1 and cerebellum activation in the “hand-raising” task is consistent with previous similar research protocols (Pfurtscheller and Neuper, 1997; Soddu et al., 2009). The SMA is implicated in the planning of motor actions and bimanual control, especially actions that are under internal control (Sakai and Passingham, 2003). The M1 works together with the premotor areas to plan and execute movements. The cerebellum plays an important role in motor control: it does not start movement, but contributes to coordination, precision, and accurate timing (Pfurtscheller and Neuper, 1997; Stoodley et al., 2012). In addition to these regions, increased activation of ACC was also found in both the patients and in the healthy control group. It has been suggested that ACC is involved in control functions, such as preparatory set and signaling cognitive conflict (MacDonald, 2000). These findings indicate a distinct need to consider the capacity for covert cognitive processing beyond overt behaviors when conducting diagnostic behavior assessment. Determining the patients’ degree of consciousness is important for optimal clinical management, and could maximize the quality of life of this population.

**TABLE 3:** Shows brain activation area related to hand raising task in 15 controls.

**Hand raising task**					

**Control**	**PMC**	**SMA**	**ACC**	**Cerebellum**	**Network status**
1	√	√	√	√	Complete
2	√	√	×	√	Partial
3	√	√	√	√	Complete
4	√	√	√	√	Complete
5	√	√	√	√	Complete
6	√	√	×	√	Partial
7	√	√	×	×	Partial
8	√	√	×	√	Partial
9	√	√	×	√	Partial
10	√	√	×	√	Partial
11	√	√	√	√	Complete
12	√	√	×	√	Partial
13	√	√	×	√	Partial
14	√	√	×	√	Partial
15	√	√	√	√	Complete

*PMC, primary motor cortex; SMA, supplementary motor area; ACC, anterior cingulate cortex. Sign “√” represents observed activation in certain brain area; Sign “×” represents certain brain area has no reaction to verbal command (*p* < 0.01, corrected).*

When in the scanner, patients were asked to raise their hand without being instructed about which hand or whether to raise both hands. This design was different from other studies in which the patients needed to raise their right or left hand precisely as the instructions were presented (Bekinschtein et al., 2011). We thought that choosing the correct hand might result in internal conflict, and of course it would increase the difficulty of the task. In the study by Bekinschtein et al. they used an fMRI paradigm to evaluate language comprehension of the residual five patients. However, this setup resulted in the exclusion of several patients. As we know, DOC patients’ wakefulness and awareness are inconsistent (Gosseries et al., 2014). As fMRI assessments cannot be taken as an exclusion tool, we need to repeat our neuroimaging and behavior assessment in these patients (Osborne et al., 2015). Recently, a small cohort hand-squeezing imagery study was performed, in which DOC patients were firstly asked to choose a proper hand (right), then followed the auditory instruction to complete the motor imagery task. Although one out of seven VS/UWS patients demonstrated they could follow the command on the hand-squeezing paradigm, the difficulty of the task may lower the possibility of detecting positive activation, especially when applied to a large number of patients (Bodien et al., 2017). Cruse et al. applied a novel EEG paradigm involving motor imagery to detect command following, which is a widely accepted clinical indicator of awareness, in 16 VS/UWS patients with absence of overt behavior. They found that three patients could repeatedly and reliably generate appropriate EEG responses to the special commands (Cruse et al., 2011). Naro et al. performed an EEG study with three motor tasks related to mirror neuron system (MNS) activation (movement observation, movement execution, and passive motor imagery of a movement) in 11 UWS and 9 MCS patients recently, they found all MCS patients and one UWS patient demonstrated an event-related synchronization in the gamma range over left frontal regions, with high Granger Casualty Index (GCI) values, in the passive motor imagery condition, similar findings can also be found in all healthy controls (Naro et al., 2018). Harrison et al. examined simultaneous EEG and fMRI in healthy controls during different mental imagery tasks, to determine whether EEG and fMRI converge upon the same conclusions about individual imagery performance, to assess their potential utility for the detection of awareness in DOC accordingly. Although robust activation could be found in most of the subjects for both EEG and fMRI, the correspondence between fMRI and EEG is not perfect. In fMRI, activation during mental arithmetic showed in 12 out of 13 subjects, surpassed any of the other imagery tasks. Mental arithmetic also produced the highest detection rates in EEG at 11/13. Contrary to the fMRI findings (running imagery task, detection rate at 2/13), EEG results for running imagery were as high as mental arithmetic. This study showed that both techniques are approximately equally able to detect consistently robust activation in mental arithmetic task, the EEG method may be more effective when detecting weaker activation (Harrison et al., 2017). In recent years, there were more studies shifted from using fMRI to EEG for detecting awareness in the field of DOC, but we couldn’t just make an easy conclusion that EEG would replace fMRI for practical reasons. We found that the results of above EEG and fMRI studies were similar to ours, despite the difference in the research protocols; we hypothesize that our paradigm might have been somewhat easier to execute than these special imagery tasks, which requires a high working memory load and extra training before data acquisition. As a result, we suggest that the hand-raising task could be taken as a clinical ancillary neuroimaging assessment in addition to behavioral assessment, and its adaption from fMRI to EEG may be an exciting idea in our future study.

Some researchers may claim the word “hand” could have automatically triggered the patterns of activation observed in the motor related areas (M1, SMA, ACC, and cerebellum). However, several reasons as follow show that this simple active paradigm could be taken as a conscious sign in DOC patients, especially in VS/UWS patients. First, if the word “hand” could elicit wholly automatic neural response without need of conscious aware, this response should transient, this transient response could not lasting 12 s (task time). At the end of task period, a “stop raising your hand” command was also given, then no active BOLD signal changes could be found in 18 s resting period, moreover, this study used block design, the same block repeated six times. Second, the responses in the patient were observed, not in brain regions that are known to be involved in word processing but in regions that are known to be involved in the motor-related tasks that the patients were asked to carry out. Third, if the “hand” automatic activation guess is right, this hand raising should be more sensitive than other task, such as own name, but we only found 2 VS/UWS patients and 2 MCS patients who followed the command in the scanner, far more less than passive own name task. All these proofs above are impossible to be explained in terms of automatic brain process.

However, command-following tasks have several inherent limitations. These paradigms require multiple cognitive systems participation (auditory, language, motor preparation, and working memory), and the reason of brain response absence could be damage or lack of recruitment of one or more of these systems. These systems’ function can be influenced by anxiety caused by noise (from MR scanner) and incomplete arousal and awareness. MRI data quality can also affect the results of motor imagery study, such as head motion and brain structural abnormalities.

## Conclusion

Our results show that despite fulfilling the clinical criteria for a diagnosis of VS/UWS and MCS, four patients retained the ability to follow verbal command and to voluntarily perform the task through brain activations rather than through speech or actual movement. It is important to mention that none of the patients physically raised their hand in the scanner. Our approach might permit an easier and more direct way to assess whether behaviorally diagnosed patients in VS/UWS are truly aware or not, and to further avoid the VS/UWS misdiagnosis. Our paradigm thus seems a possible supplementary tool to diagnose some patients who are inner conscious but unable to produce an overt motor output.

## Data Availability

All datasets generated for this study are included in the manuscript.

## Ethics Statement

Written informed consent was obtained from the legal representatives of all the patients and all the healthy volunteers. The study was approved by the Ethics Committee of Hangzhou Normal University School of Medicine, Hangzhou, China.

## Author Contributions

FW and HD were responsible for the writing and processing of the experiment. NH, LH, and AT helped with editing the manuscript. XH and SJ managed the patients during the experiment. WH, YY, and JW assist the experiment. CS and SL provided guidance on experimental design and the data analysis.

## Conflict of Interest Statement

The authors declare that the research was conducted in the absence of any commercial or financial relationships that could be construed as a potential conflict of interest.
